# Antibody-Antineoplastic Conjugates in Gynecological Malignancies: Current Status and Future Perspectives

**DOI:** 10.3390/pharmaceutics13101705

**Published:** 2021-10-15

**Authors:** Cristina Martín-Sabroso, Irene Lozza, Ana Isabel Torres-Suárez, Ana Isabel Fraguas-Sánchez

**Affiliations:** 1 Department of Pharmaceutics and Food Technology, Faculty of Pharmacy, Complutense University of Madrid, 28040 Madrid, Spain; crmartin@ucm.es (C.M.-S.); lozza.1584043@studenti.uniroma1.it (I.L.); galaaaa@ucm.es (A.I.T.-S.); 2 Institute of Industrial Pharmacy, Complutense University of Madrid, 28040 Madrid, Spain

**Keywords:** auristatins, cervical cancer, endometrial cancer, maytansinoids, mirvetuximab soravtansine, ovarian cancer, sacituzumab govitecan, targeted-chemotherapy, trastuzumab deruxtecan

## Abstract

In the last decade, antibody-drug conjugates (ADCs), normally formed by a humanized antibody and a small drug via a chemical cleavable or non-cleavable linker, have emerged as a potential treatment strategy in cancer disease. They allow to get a selective delivery of the chemotherapeutic agents at the tumor level, and, consequently, to improve the antitumor efficacy and, especially to decrease chemotherapy-related toxicity. Currently, nine antibody-drug conjugate-based formulations have been already approved and more than 80 are under clinical trials for the treatment of several tumors, especially breast cancer, lymphomas, and multiple myeloma. To date, no ADCs have been approved for the treatment of gynecological formulations, but many formulations have been developed and have reached the clinical stage, especially for the treatment of ovarian cancer, an aggressive disease with a low five-year survival rate. This manuscript analyzes the ADCs formulations that are under clinical research in the treatment of gynecological carcinomas, specifically ovarian, endometrial, and cervical tumors.

## 1. Introduction

The term “gynecological cancer” designates a group of neoplasms that occur in or on female reproductive organs and genitals, including carcinomas of the vagina, vulva, cervix, uterus, ovaries, and fallopian tubes. These neoplasms affect millions of women worldwide across all ages, impairing their health and quality of life and triggering, in many patients, an early death. The American Cancer Society estimated 116,760 new cases of gynecological malignancies and 34,080 deaths in the United States in 2021. Among all of them, uterine (including endometrial cancer and sarcomas of the uterus), ovarian and cervical carcinomas are the most frequent neoplasms, representing around the 57, 18 and 12% of the new estimated cases, and around the 37, 40 and 12% of the cancer-related deaths respectively [1], constituting major health problems.

The treatment of gynecological tumors depends on the cancer type and disease stage. In all cases, surgery represents the mainstay treatment option [2], usually, in combination with chemotherapy, that is of particular interest in advanced stages of the disease and in invasive carcinomas such as ovarian tumors [3,4], and, in some cases, radiotherapy. Several immunotherapeutic treatments, including immune checkpoint inhibitors and monoclonal antibodies, have also been approved in the last few years for the treatment of gynecological malignancies, especially for uterine, cervical, and ovarian carcinomas, improving the therapeutic options of these diseases [5,6,7].

**Figure 1:**
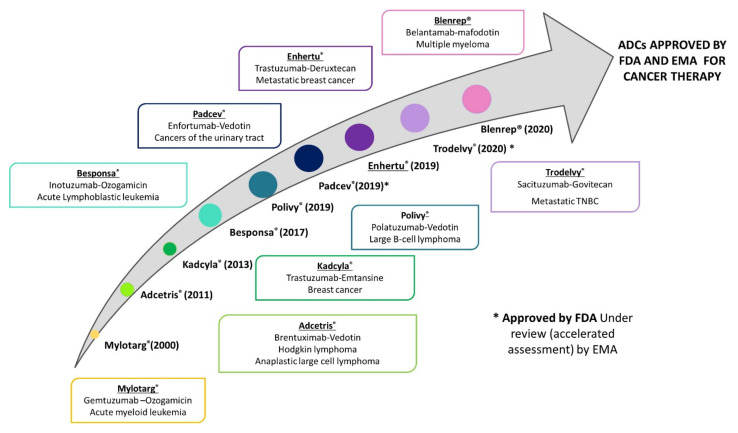
Timeline of the ADCs that have been approved by the Food and Drug Administration (FDA) and the European Medicine Agency (EMA) and are currently available in USA and the European Union for cancer therapy.

A particular case of interest is the use of monoclonal antibodies as they can be conjugated with antineoplastic agents. This allows to get a selective delivery of the chemotherapeutic drug at the tumor level, and, consequently, to improve the antitumor efficacy and, especially to decrease chemotherapy-related toxicity. It should be noted that some antibodies have also an antitumor effect per se, increasing the anticancer effect of the antineoplastic [8,9]. To date, nine antibody-drug conjugates (ADCs) have been approved for cancer therapy (Figure 1), and 80 other formulations are under clinical investigation [10]. In the case of gynecological malignancies, there are no approved ADCs. However, many of them are under clinical trials, especially for the treatment of ovarian cancer [11].

This review will focus on the ADCs designed for the treatment of gynecological malignancies, specifically for the treatment of endometrial, ovarian, and cervical, analyzing the formulations that have reached the clinical investigation.

## 2. ADCs in Gynecological Tumors: Structure and Function

**Figure 2:**
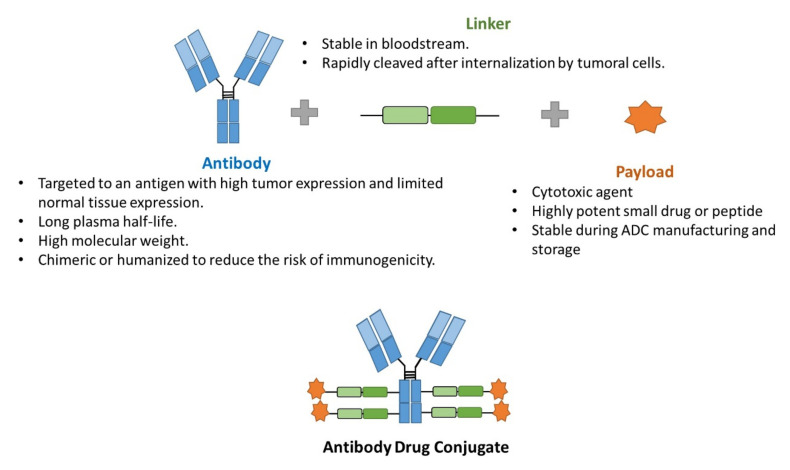
Scheme of the general structure of an ADC.

ADCs may represent an ideal treatment chemotherapeutic option in cancer disease: selective and effective with cancer cells. They combine the tumor-selective activity of monoclonal antibodies and the effective cytotoxic effect of chemotherapy with a lower rate of the undesirable side effects of antineoplastics, and, in some cases, a higher efficacy, due to tumor specificity. They consist of three components: (i) a tumor antigen-selective monoclonal antibody, (ii) a potent antineoplastic agent, and (iii) a linker that binds these two entities (Figure 2) [12,13]. Regarding the general mechanism of action, ADCs bind to their specific target that is expressed on the surface of cancer cells, and are internalized by a receptor-mediated endocytosis. Then, the antineoplastic drug is released and exerts its anticancer activity [12,14]. Some antibodies have also an antitumor effect per se which improving the overall antitumor efficacy [15]. This is the case of trastuzumab, approved for the treatment of human epidermal growth factor receptor-2 (HER-2) positive breast and gastric tumors [16], and that it is present in two approved ADCs: Kadcyla^®^ and Enhertu^®^. Trastuzumab exerts its anticancer effect by inhibiting the proliferation induced by HER-2, as when it binds to these receptors, it blocks its auto-activation and promotes its degradation, and by activating antibody-mediated cytotoxicity [17].

An ideal ADC should contain a monoclonal antibody targeted to a tumor antigen solely expressed or overexpressed on the surface of cancer cells and with a low expression on the healthy tissues to get selective location, and consequently, a specific cytotoxic effect at the tumor level. In the case of gynecological tumors, several antigens have been exploited (displayed in Table 1). For example, in the case of ovarian cancer, several ADCs target folate receptor α (FRα), which is overexpressed in 90% of ovarian tumors [18], and represents an excellent target to get a selective location of the drugs at ovarian cancer cells. MUC16, also known as the cancer antigen CA125, is another exploited target. This is a glycoprotein involved in tumor metastases of ovarian cancer. In fact, it is the most commonly used serum biomarker for the diagnosis of epithelial ovarian cancer as its levels are related to the disease progression [19,20]. Mesothelin is another exploited target in this neoplasm. It is a tumor differentiation antigen overexpressed on ovarian cancer cells, that participates in apoptosis evasion, tumor invasion and peritoneal metastases of this carcinoma [21,22]. It is also being evaluated as a tumoral target in fallopian tubes cancers. In the case of endometrial carcinomas, most parts of evaluated ADCs target HER-2 which is involved in the proliferation, differentiation, migration, and survival of cancer cells and is overexpressed in this type of tumor [23,24,25].

Regarding the cytotoxic payloads, the candidates must comply with several requirements. First, the payload must allow the conjugation with the monoclonal antibody in aqueous buffers. Consequently, they must have an appropriate water solubility and stability in these buffers [49]. Second, they must be potent antineoplastics, with a concentration inhibitory 50 value in the subnanomolar range [44,50], as although ADCs are highly selective to tumoral cells, just a small fraction of the drug reaches the intracellular target [51]. In fact, a study undertaken in xenograft tumor models in mice with radiolabeled antibodies demonstrated that low than 1% of the injected antibody accumulates per gram of tumor [52]. Moreover, it should be considered that the number of cytotoxic molecules that are attached to each antibody is limited, as a high drug-to antibody ratio may increase the risk of toxicity and limit the binding of the monoclonal antibody to its target and, consequently, the efficacy of the ADC [53], being necessary the use of potent drugs. Third, the payloads should be small to decrease the risk of immunogenicity and fourth, they must have sufficient stability in plasma considering the long-circulation time of the monoclonal antibodies [54].

It should be mentioned that most of the used payloads target DNA or tubulin [49]. This is the case of auristatins, maytansinoids and calicheamicins, common drugs used in the development of ADCs. In the case of the ADCs developed for the treatment of gynecological malignancies, some of the most used drugs are monomethyl auristatin E, and ravtansine (DM4). Monomethyl auristatin E (MMAE) is a synthetic derivate of dolastin, a peptide isolated from the sea hare Dolabella auriculari [55]. This cytotoxic agent targets tubulin, inhibiting its polymerization, and consequently, blocking the mitosis [56]. Despite their potent anticancer effect, this drug shows high toxicity in its native form, being used as an ADC payload. In fact, MMAE complies with all the requirements of an ideal ADC payload: high potency, water-solubility, physiological stability and suitability for the conjugation [57], being used in a large number of ADCs. DM4 is a thiol-bearing maytansinoid that contains methyl disulfide substitutions at the C3 N-acyl-N-methyl-L-alanyl ester side chain of maytansine, being suitable for monoclonal antibody attachment [58]. It exerts its anticancer activity by inhibiting tubulin assembly into microtubules and arresting cell cycle in the G2/M phase [59]. Finally, topoisomerase I inhibitors are also used. This is the case of SN-38, the active metabolite of irinotecan (CPT-11), a camptothecin analog, that exerts a cytotoxic effect by inhibiting DNA topoisomerase I [60]. SN-38 is very potent, showing around 1000-fold potency than CPT-11. Due to this high cytotoxicity, SN-38 is too toxic, producing delayed serious diarrhea, with or without neutropenia and myelosuppression [61], and cannot be administered as a free drug. It should be noted that SN-38 shows a low aqueous solubility (11–38 μg/mL) [61]. However, it is a suitable molecule to be attached to monoclonal antibodies through usual conjugation methods [49].

Antibody-drug conjugates may incorporate active compounds other than antineoplastics ones as payload, such as immunostimulatory agents, developing immune-stimulating antibody conjugates. In this case, the main advantage of these formulations consists of the increase of the effect due to the selective accumulation of these compounds at tumoral cells [62].

**Figure 3:**
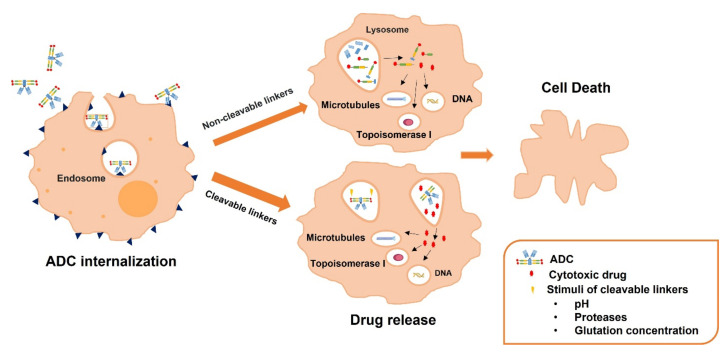
Scheme showing the general release mechanism of antineoplastic drugs from ADCs containing non-cleavable and cleavable linkers. After ADC internalization, when a cleavable linker has been used, the cytotoxic drug release in the lysosomes is triggered by: (i) low pH of values of the intracellular compartment in acid-labile linkers, (ii) the presence of proteases in protease-cleavable linkers and (iii) the high glutathione concentrations in disulphide-linkers. However, when a non-cleavable linker has been used the lysosomal proteolytic machinery metabolizes the monoclonal antibody producing the release of the cytotoxic drug bound to the linker and the amino acid appendages.

Regarding linkers, they are used to connect the payload molecules to the monoclonal antibody, through their disulphides, hydrazones, and thioethers groups, influencing the stability of the ADC [12,62]. The used linkers should comply with two requirements: to have sufficient stability to allow prevent premature cleavage in the bloodstream and to be rapidly cleaved after ADC internalization to release the antineoplastic [63,64,65]. There are two different categories based on the release mechanism of the antineoplastic: cleavable and non-cleavable linkers. The release mechanism of cleavable linkers is based on several characteristics of the physiological environment: (i) low pH values of the intracellular compartment compared to the neutral pH of the extracellular compartment (acid-labile linkers), (ii) the presence of proteases that release the drug at the area of junction (protease-cleavable linkers) and (iii) the high glutathione concentrations that reduce disulphide bridges (disulphide-linkers) [66]. On the contrary, for non-cleavable linkers, there is no inbuilt chemical trigger to break the linker and release the cytotoxic payload. After ADC internalization in the target cells, the monoclonal antibody is metabolized by the lysosomal proteolytic machinery into aminoacids, triggering the release of the antineoplastic bound to the linker and the amino acid appendages [67] (Figure 3). It should be considered that the ADCs developed using non-cleavable linkers are more stable. Moreover, this release mechanism prevents a premature drug release, reducing systemic toxicity [68]. Nevertheless, it is required and efficient internalization and lysosomal trafficking. This release mechanism could also affect the drug effect, specially comparing with the use of cleavable linkers. In fact, the most used linkers in ADCs are cleavable linkers: acid-cleavable linkers, protease-cleavable linkers (specially cathepsin cleavable-linkers), and disulfide-containing-reducible linkers [69].

The selection of the linker depends on the antineoplastic payload, the monoclonal antibody, and the antigen target. For example, in the case of more hydrophobic drugs, the use of more hydrophilic linkers may limit the activity of the efflux pumps, that trend to take these drugs out of the tumoral cells, overcoming the resistances related to this mechanism and improving the anticancer activity if these antineoplastics [53].

Finally, it is worth mentioning that some ADCs show a bystander killing effect that occurs when the antineoplastic agents not only act in the targeted cells but also in the surrounding or bystander cells, that can express or not the target antigen [70]. This is especially interesting as the anticancer effect is potentiated. For example, while Enhertu^®^, a monoclonal antibody that consists of trastuzumab bound to deruxtecan via a cathepsin cleavable linker, has this bystander killing activity, Kadycla^®^ also known T-DM1 and consisting of trastuzumab bound to emtansine using a non-cleavable linker, does not show this effect [71]. This difference could be attributed to the cytotoxic payload but also to the chemistry of the linker [72,73], indicating the importance of a good design of the ADC.

## 3. ADCs for Ovarian Cancer

Ovarian cancer is the most lethal gynecological malignancy due to its late diagnosis and high recurrence [74], showing a low five-year survival rate of around 45% [75]. Currently, the first-line treatment consists of surgery and chemotherapy (mainly based on platinum compounds). Several targeted therapies, including (poly-ADP-ribose polymerase) (PARP) inhibitors and bevacizumab, a monoclonal antibody targeting vascular endothelial growth factor, have been approved for ovarian cancer therapies in the last decade [76,77], improving the outcome of this disease. However, ovarian cancer treatment is still challenging. In this way, ADCs can be a good therapeutic option. To date, no ADC formulation has been approved for this neoplasm. However, several formulations have reached the clinical investigation and have been or are being evaluated in ovarian cancer patients (Table 2) [78].

### 3.1. ADCs Containing Maytansinoids

As previously mentioned, maytansinoids, inhibitors of the tubulin polymerization, are good candidates for the development of ADCs. In fact, Kadcyla^®^ is an ADC approved for early [80,81] and advanced [82] breast cancer therapy in both Europe and the USA that contains a maytansinoid (DM-1) as payload [45]. In the case of ovarian cancer, three maytansinnoid based formulations are under clinical research: (i) Mirvetuximab soravtansine, (ii) Praluzatamab ravtansine, and (iii) Anetumab ravtansine.

#### 3.1.1. Mirvetuximab Soravtansine

Mirvetuximab soravtansine, also known as IMGN853, is an ADC that consists of M9346A, a monoclonal antibody targeted to FR-α, bound to the antineoplastic drug DM-4 (also known as soravtansine or ravtansine) through a cleavable disulfide linker [26,27]. This formulation has reached the phase III clinical stage in ovarian cancer patients, being the most advanced ADC formulation in this neoplasm.

A phase I study undertaken in 43 patients with solid tumors, including ovarian, non-small cell lung, endometrial and renal cancer among others, demonstrated that mirvetuximab soravtansine showed an acceptable safety profile when administered at doses of 0.15 to 7.0 mg/kg, being fatigue, blurred vision, and diarrhea, classified as mild to moderate in severity, the most common adverse effects. Severe hypophosphatemia and punctate keratitis were detected at doses of 5 mg/kg and 7 mg/kg respectively. Regarding the clinical efficacy, a benefit in terms of partial response or stable disease was detected in 22 patients, most of them diagnosed with ovarian cancer [83]. The clinical efficacy could be related to the expression of FR-α, more than usual in this neoplasm. In fact, a study carried out in ovarian cancer patients showed that the clinical efficacy of this ADC was correlated with the expression of these receptors at the tumoral cells [84].

The efficacy of this formulation in combination with other anticancer drugs has also been evaluated. A small study carried out in 18 patients with relapsed ovarian cancer reported that this ADC at doses of 5–6 mg/kg was relatively safe and effective in combination with carboplatin, with an overall response rate of 71%. Nausea, diarrhea, thrombocytopenia, blurred vision, and fatigue were the most common adverse effects [85]. Similar results in terms of toxicity were detected with the combination of mirvetuximab soravtansine (at doses of 6 mg/kg) and bevazizumab or pembrolizumab. However, in these studies, the overall response rate was much lower (around 50%) [86,87]. Finally when mirvetuximab soravtansine was combined with carboplatin and bevazizumab a high overall response rate (around 81%) was detected, with an acceptable safety profile [88]. In fact, a clinical trial (NCT04274426) has been recently launched to confirm the promising combination of this ADC (administered at 6 mg/kg once every three weeks) and carboplatin comparing to carboplatin plus other conventional antineoplastics (paclitaxel, liposomal doxorubicin, and gemcitabine).

A recent phase III study (NCT02631876) carried out in 366 patients with ovarian cancer demonstrated a clinical benefit of mirvetuximab soravtansine at doses of 6 mg/kg compared to chemotherapy (paclitaxel, pegylated liposomal doxorubicin, or topotecan). On the one hand, in terms of toxicity, a lower incidence and grade in severity were detected in the ADC-treated patients. On the other hand, in terms of efficacy, although a significant improvement in progression-free survival neither mirvetuximab soravtansine nor chemotherapy-treated patients was appreciated, the responses rates were higher in the patients treated with the ADC [89].

#### 3.1.2. Praluzatamab Ravtansine

**Figure 4:**
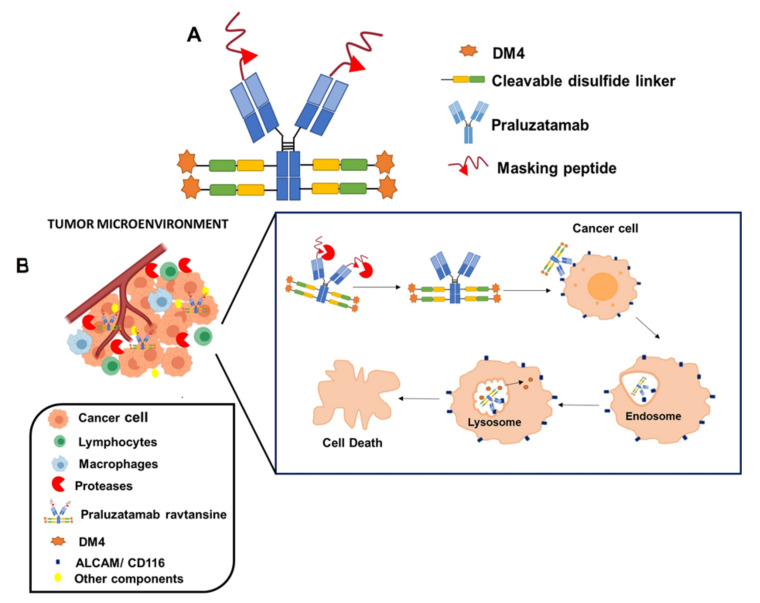
Structure (**A**) and mechanism of action (**B**) of Praluzatamab ravtansine.

Praluzatamab ravtansine, also known as CX-2009, is another developed ADC that contains DM4 as payload, in this case, linked, via a cleavable disulfide linker, to praluzatamab, a monoclonal antibody targeting the activated leukocyte cell adhesion molecule (ALCAM/CD116), a glycoprotein overexpressed by certain tumors and that is involved in tumor progression [28]. This formulation is of special interest from a structural point of view. With the aim of avoiding the binding of this monoclonal antibody to the CD116 antigen expressed in healthy cells, the binding area of this antibody is masked by a cleavable peptide, that is degraded by tumor-associated proteases that are specifically found in the tumor microenvironment. Then, the monoclonal antibody binds to the CD116 antigen of the surface of tumoral cells, the ADC is then internalized and DM4 payload is released intracellularly (Figure 4). A study undertaken in a mouse model of lung cancer overexpressing CD116 antigen with ^89^Zr-radiolabeled formulation has demonstrated a selective targeting at the tumoral level and a reduced accumulation at healthy tissues, indicating that this masking strategy is useful to decrease the toxicity and to improve the efficacy of this formulation [90]. This ADC has been mainly evaluated in breast cancer patients. However, a study carried in patients with several types of advanced carcinomas, also included ovarian cancer patients (NCT03149549). Preliminary results of this study demonstrated a partial response in ovarian cancer-treated patients, just 9% of the women demonstrated a partial response, indicating a poor effect in this neoplasm. Regarding the toxicity, the formulation was in general well tolerated, being fatigue, nausea, vomiting, arthralgias, and infusion-related reactions were common adverse effects. Ocular toxicities were also detected in some patients [91]. It should be mentioned that this study included a small cohort of ovarian cancer patients, and further investigation is probably needed to evaluate verify the potential utility of praluzatamab ravtansine in advanced ovarian tumors, especially considering the lack of effective treatments for this disease.

#### 3.1.3. Anetumab Ravtansine (BAY 94-9343)

Anetumab ravtansine, also known as BAY 94-9343, is another DM-4-based ADC targeted to mesothelin, a surface glycoprotein overexpressed in all mesotheliomas and in ovarian and pancreatic tumors, and with a limited expression in healthy tissues [29,30]. In this formulation, the maytansinoid DM-4 is conjugated to an anti-mesothelin-monoclonal antibody via a disulfide reducible linker.

This antibody-drug conjugate has demonstrated a potent selective activity against mesothelin overexpressing tumors, with an inhibitory concentration 50 of around 0.72 nM. In vivo, it not only demonstrated induction of the death of mesothelin-positive tumoral cells but also to exert a bystander effect on neighboring mesothelin-negative cancer cells [92]. Moreover, it also demonstrated an additive effect with copanlisib, bevacizumab and conventional antineoplastics such as cisplatin and doxorubicin [93].

A phase I clinical trial (NCT01439152), undertaken in patients with mesothelin overexpressing tumors, including patients with ovarian cancer, demonstrated a maximum tolerated dose of anetumab ravtansine of 2.2 mg/kg once per week or 6.5 mg/kg every three weeks. In general, the formulation, administered using this protocol, was well tolerated, being gastrointestinal disorders, fatigue, peripheral sensory neuropathy, anorexia, and keratitis the most common adverse effects. Regarding the efficacy, more than 50% of the treated patients respond to the treatment, exhibiting a stable disease or a partial response [94]. Preliminary results have also demonstrated that the combination of this formulation and liposomal doxorubicin could be a potential therapeutic alternative in platinum-resistant-ovarian cancer patients (NCT02751918). In general, this combination was safe and well-tolerated, without any dose-limiting toxicity, and an overall response rate of around 42% in patients with high mesothelin-expressed tumors [95].

### 3.2. ADCs Containing Auristatins

Auristatins are also excellent payloads for the development of ADCs, being MMAE and MMAF the most used. Numerous ADCs containing these agents as antineoplastics have been developed. To date, there are 4 ADCs that contain auristatins as payload (Adcetris^®^, Polivy^®^, Padcev^®^, and Blenrep^®^) that are approved by FDA and/or EMA for the treatment of lymphomas, multiple myeloma, bladder cancer or tumors of the urinary tract) [96,97]. Many other formulations are under clinical research, including formulations for ovarian cancer: (i) DMUC4064A, (ii) Sofituzumab vedotin, (iii) Tisotumab vedotin, (iv) DMOT4039A, (v) Enapotamab vedotin, (vi) Lifastuzumab Vedotin, (vii) Cofetuzumab pelidotin and (viii) Upifitamab rilsodotin.

#### 3.2.1. DMUC4064A

DMUC4064A is an ADC consisting of two molecules of MMAE linked to an anti-MUC16 monoclonal antibody via a protease labile linker. When administered at doses of 5.2 mg/kg (NCT02146313), it has demonstrated a promising anticancer effect in patients with platinum-resistant ovarian cancer, showing an overall response rate of around 45%. The safety profile was manageable, being ocular toxicities (specifically blurred vision and dry eyes) the most common adverse effects, appearing in 75% of the patients. However, in most of the cases, they were solved by decreasing the dose or by using re-wetting or steroid doses. Other treated patients also experienced fatigue, nausea, diarrhea, and peripheral neuropathy [31].

#### 3.2.2. Sofituzumab Vedotin

Sofituzumab vedotin, also known as DMUC5754A, is another ADC formulation containing MMAE as payload, linked to anti-MUC-16 antibody via a cathepsin cleavable linker. A study undertaken in 66 patients with platinum-resistant ovarian cancer demonstrated a low response rate of around 7% (NCT01335958) that could be related to the expression of MUC-16, as only those patients that show a high expression to this ovarian tumor-associated antigen responded to the treatment. As with DMUC4064A, this ADC also produced fatigue, nausea, diarrhea, and peripheral neuropathy. However, no ocular-related toxicities were appreciated, indicating that this toxicity was related to the formulation, and underlying the importance of the linker and the conjugation strategy [32].

#### 3.2.3. Tisotumab Vedotin

Tisotumab vedotin, also named Humax^®^-TF, is an antibody-drug conjugate based on MMAE linked, via cathepsin cleavable linker, to a monoclonal antibody targeting tissue factor (TF), a transmembrane receptor and cofactor for factor VII/FVIIa [33], expressed by fibroblasts and epithelial cells and overexpressed in many tumors [98], including ovarian tumors [99]. In fact, a study carried out in ovarian cancer women, reported an overexpression of this receptor in more than 75% of the patients [100], suggesting their utility as a target. A phase I dose-escalation study (NCT02001623) carried out in patients with different types of solid tumors, including ovarian cancer demonstrated a maximum tolerated dose of 2 mg/kg, as at higher doses (2.2 mg/kg) this formulation exhibited serious dose-limiting toxicities: mucositis, type 2 diabetes mellitus, and neutropenic fever. A phase II dose expansion study demonstrated that epistaxis, fatigue, and gastrointestinal alterations were the most frequent adverse effects related to tisotumab vedotin when administered at doses of 2 mg/kg. Regarding the efficacy in the ovarian cancer group, an objective response was detected in only 13.9% of the patients [101], suggesting that although ovarian tumors trend to overexpress TF receptors, a higher accumulation was not achieved.

#### 3.2.4. DMOT4039A

DMOT4039A is another ADC containing MMAE, in this case, linked to an anti-mesothelin monoclonal antibody via a protease cleavable linker. This formulation, when administered at doses in the range of 2.4–2.8 mg/kg, has demonstrated a tolerable safety profile, being gastrointestinal toxicities the most common adverse effects, and antitumor activity in 30% of the treated patients (NCT01469793) [79].

#### 3.2.5. Enapotamab Vedotin

Enapotamab vedotin, also known as Humax-AXL or AXL-107-MMAE, is an ADC formulation in which MMAE is conjugated to an anti-AXL-monoclonal antibody through a cathepsin cleavable linker. AXL is a member of the TAM (TYRO3, AXL and MER) family of tyrosine kinase receptors which are overexpressed in tumors and involved in the proliferation, invasion, and metastasis of tumoral cells. Their expression has been related to chemoresistance and poor prognosis [34]. In fact, it has been associated with taxane and platinum resistances in ovarian cancer [102]. A phase I-II clinical trial is underway to evaluate the toxicity and establish the maximum tolerated dose in patients with solid tumors, including patients with ovarian cancer and other gynecological malignancies such as cervical and endometrial cancer (NCT02988817). No data concerning its efficacy or toxicity have yet been reported.

#### 3.2.6. Lifastuzumab Vedotin

MMAE has also been conjugated to a monoclonal antibody targeting the sodium-dependent phosphate transport protein 2b (NaPi2b), which is involved in the active transport of phosphate into cells [35], through an enzymatically cleavable linker. NaPi2b is overexpressed in many cancer types, including ovarian tumors. In fact, a study demonstrated a high expression in around 70% of the carcinomas of the ovary [103]. This formulation is known as lifastuzumab vedotin or DNIB0600A.

A phase I study (NCT01911598) carried out in patients with platinum-resistant ovarian cancer and non-small cell lung carcinoma reported that lifastuzumab vedotin was, in general, well-tolerated when administered at doses in the range of 0.2–2.8 mg/kg, without reaching dose-limiting toxicities. Nausea, vomiting, fatigue, decreased appetite and sensory neuropathy were the most common adverse effects. Regarding the efficacy, while only a small effect was appreciated in patients with lung cancer, a partial response was detected in 46% of ovarian cancer patients, suggesting a promising effect in this neoplasm. A dose of 2.4 mg/kg once every three weeks was recommended based on the reported adverse effects and clinical response [104]. However, the response duration was relatively short, without any statistically significant improvement compared to liposomal doxorubicin [105], indicating that a further evaluation should be done.

Finally, the combination lifastuzumab vedotin with carboplatin and bevacizumab showed a promising anticancer activity in patients with platinum-resistant ovarian cancer, reporting partial or complete responses in around 60% of the patients and a median survival rate of around 10 months. Although the safety profile of this combination was manageable, severe side effects (neutropenia and thrombocytopenia were the most common) were detected in 82% of the treated patients [106], suggesting that further studies are necessary to verify the clinical benefit of this combination, especially considering the high incidence of severe adverse effects.

#### 3.2.7. Cofetuzumab Pelidotin

Cofetuzumab pelidotin, also known as PF-06647020 or ABBV-647, is an ADC containing Auristatin-0101 as payload linked, through a cathepsin cleavable linker, to a monoclonal antibody targeted to protein tyrosine kinase 7 (PTK7) [36], that is upregulated in several cancer types, including colon, breast, and esophageal tumors among others. In ovarian cancer, this protein has been found overexpressed in the 45% of the tumors. However, it should be mentioned that the higher tumor stage the lower expression, suggesting that it could be a good prognostic marker in this pathology [107]. Despite this variable expression, this formulation has been evaluated in ovarian cancer patients (NCT02222922), showing a response rate of around 27% when administered every two or three weeks at doses in the range of 0.2–3.7 mg/kg. It should be highlighted that responders tended to have a high or intermediate expression of PTK7, indicating that the low overexpression of this target could explain the low response rate. Nausea, alopecia, fatigue, headache, neutropenia, and vomiting were the most common adverse effects related to this ADC [108].

#### 3.2.8. Upifitamab Rilsodotin

Upifitamab rilsodotin, also known as XMT-1536, is an ADC that consists of an anti-, NaPi2b monoclonal antibody, linked to dolaflexin, which consists of a hydrophilic polymer conjugated to the precursor of MMA-F (auristatin F-hydroxypropylamide). This conjugation strategy considerably increased the cytotoxic payload. Usually, when the drug is directly conjugated to the monoclonal antibody, has an average of 3–4 payload molecules per antibody, but using dolaflexin technology a loading of 10–15 auristatin molecules per antibody is obtained [37,38]. The excision of the polymer and the drug occurred intracellularly into the tumoral cells, where the precursor of auristatin F is metabolized to the active compound and exerts its anticancer activity [37].

Preliminary results of a phase I study (NCT03319628), undertaken in patients with ovarian cancer, demonstrated that this ADC was in general well tolerated when administered in the range of 36–43 mg/kg every four weeks, without detecting dose-limiting toxicities. Fatigue, nausea, vomiting, pyrexia, decreased appetite, diarrhea, and transient increase in aspartate transaminase were some detected adverse effects. A positive response, in terms of partial response and stable disease, was detected [109,110], indicating the promising utility of this formulation in ovarian cancer therapy. In fact, a phase I-II clinical trial has been recently launched to evaluate its combination with carboplatin in patients with serious grade ovarian cancer (NCT04907968).

### 3.3. ADCs Containing Calicheamicins

The term “calicheamicins” designates a family of enediyne antitumor antibiotics isolated from the fermentation broth of Micromonospora echinospora. They exert their action by binding DNA in the minor groove and triggering DNA strand scission [111,112]. They show a potent antitumor activity, being suitable candidates as ADC payloads. In fact, two ADCs: Mylotarg^®^ and Besponsa^®^, already approved for the treatment of acute myeloid leukemia and acute lymphoblastic leukemia respectively, contain calicheamicins as antineoplastics [113].

PF-06647263 is an ADC formulation consisting of calicheamicin conjugated to a monoclonal antibody targeting tyrosine kinase ephrin receptor A4 (EFNA-4) through a hydrazone cleavable linker. Preclinical studies have reported that it was able to induce suppression of triple-negative breast and ovarian carcinomas [39]. However, a clinical phase I study (NCT02078752) carried out in patients with solid tumors, mainly breast and ovarian carcinomas, resistant to conventional therapies reported a limited efficacy, as just 10% exhibited a slight tumor reduction, in terms of partial response. Twenty-two percent of the patients experienced stable disease. It should be noted that response was not correlated to EFNA-4 expression levels [114], suggesting that this target, is not as good a target as expected to get a selective chemotherapy. Fatigue, nausea, diarrhea, vomiting, thrombocytopenia, and mucosal inflammation were the most common adverse effects related to this formulation.

### 3.4. ADCs Containing Pyrrolobenzodiazepines

Pyrrolobenzodiazepines, DNA cross-linking agents [115], are also good candidates for the development of ADCs. Several formulations containing these antineoplastics have reached the clinical stage in ovarian cancer patients. However, ADCs containing pyrrolobenzodiazepines have not yet been approved.

#### 3.4.1. Tamrintamab Pamozirin

Tamrintamab pamozirin, also known as SC-003, is an antibody-drug conjugate that consists of a pyrrolobenzodiazepine dimer linked to tamrintamab, a monoclonal antibody targeted to dipeptidase 3 (DPEP3 or MBD3) via a protease-cleavable maleimide linker. DPEP3 is a glycosylphosphatidylinositol-anchored metallopeptidase that can be overexpressed in ovarian tumors [40] and may be a good target antigen. However, a phase I study (NCT02539719) carried out in patients with this type of carcinomas demonstrated a low efficacy in patients with epithelial ovarian cancer, showing a low and not durable overall response rate of around 4%. The most common adverse effects were fatigue, nausea, decreased appetite, pleural effusion, abdominal pain, and peripheral edema [116]. This study indicated that tamrintamab pamozirin conjugate is not the best option to get a selective chemotherapy in ovarian cancer patients.

#### 3.4.2. SC-004

SC-004 is another pyrrolobenzodiazepine-based antibody drug conjugate that consists of a monoclonal antibody targeted to claudins 6 and 9 (CLDN6/9), tight junctional proteins, that are overexpressed in ovarian tumors [117], bound to a pyrrolobenzodiazepine dimer. Preliminary results in patients with platinum-refractory or resistant high-grade serous epithelial ovarian cancer have shown that SC-004, administered at doses of 0.005–0.3 mg/kg, has a low tolerability, similar to other pyrrolobenzodiazepine based formulations. The 88% of the patients suffered adverse effects, which were fatigue, peripheral edema, vomiting, pleural effusion, urinary tract infection, rash maculo-papular, anemia, abdominal pain, diarrhea, and decreased appetite. In 33% of the patients, grade 3 adverse effects were detected. The efficacy was also low. A partial response in the patients that overexpress claudins, was just detected in one patient. The low efficacy-toxicity balance indicates that the clinical investigation with this ADC should be discontinued (NCT03138408) [118].

### 3.5. ADCs Containing Hemiasterlin Derivates

Hemiasterlin is a natural product, member of a family of cytotoxic peptides initially isolated from marine sponges, with a potent anticancer effect, in the sub-nanomolar range, against several types of tumors, specially HER-2 positive [119], such a breast and cervix. They act by inhibiting microtubule assembly [120].

Due to their high potency, they are candidates for ADC development. However, no ADCs containing hemiasterlin derivates as payload have yet been approved. A formulation containing SC209, a hemiasterlin derivate, linked to an anti-FRα- monoclonal antibody has been developed (STRO-002). An enzymatic cleavable linker was used in this conjugation. This formulation has demonstrated an efficient targeting in mouse models of ovarian and endometrial carcinomas, as they overexpress FRα receptors, indicating that this could be a good treatment option in both neoplasms, showing tumor growth inhibition [42]. In fact, a phase I study is underway in patients with platinum-resistant ovarian and fallopian tube carcinomas (NCT03748186). Preliminary results have demonstrated that STRO-002 at doses in the range of 0–5−6 mg/kg, was relatively safe without reaching the maximum tolerated dose. Fatigue, nausea, decreased appetite and dizziness, all of them low in severity, were the most common adverse effects. Almost 50% of the patients exhibited a treatment response in terms of stable disease or partial response [121], indicating the promising use of this formulation in ovarian cancer treatment.

### 3.6. ADCs Containing Eribulin

MORAB-202 consists of eribulin, a cytotoxic agent that inhibits the growth phase of the microtubules, conjugated to farletuzumab, a monoclonal antibody targeting FRα, through a cathepsin-cleavable linker. This formulation has shown a potent pre-clinical cytotoxic effect in FRα overexpressing tumors [43].

A phase I clinical trial (NCT03386942), carried out in patients FRα positive advanced solid tumors, including patients with carcinomas of the ovary, has reported that, in general, it is well-tolerated when administered at doses in the range of 0.3 to 1.2 mg/kg once every three weeks. Mild-to-moderate adverse effects related to this ADC were detected in 95% of the patients, being neutropenia and leukopenia the most common toxicities. A patient (a total of 22 patients were enrolled in the study) also experienced severe serum alanine aminotransferase and γ-glutamyl transferase increases. Regarding to the antitumor activity, positives responses were observed in 18 patients (81%), in terms of complete response (one patient, 4.5%), partial response (nine patients, 40.5%) and stable disease (eight patients, 36%) [122]. Due to these promising results, a phase I-II clinical study (NCT04300556) has been launched in patients with solid tumors, including women with ovarian and endometrial tumors to study in depth the safety and efficacy of this formulation.

## 4. ADCs for Uterine Tumors: Endometrial and Cervical Cancer

Endometrial cancer is the most frequent gynecological neoplasm, representing around 90% of the tumors of the uterine corpus. In the last years, its incidence has considerably increased, due to, at least in part, declining rates of hysterectomy for benign causes. In general, endometrial cancer shows a high five-year survival rate of around 83% [123,124]. However, it has hardly improved in the last three decades as in the 1980s the survival rate was around 81% [125]. Cervical cancer that affects the cells lining the cervix represented a major health problem in women in the twentieth century, as it used to be one of the leading causes of cancer death in the female population. However, in the last decades due to the pre-screening of Pap tests, that allow the identification of pre-cancerous lesions, and the approval of papilloma vaccines, the incidence and mortality of this neoplasm have considerably decreased [126,127].

Surgery is the gold standard treatment for both endometrial and cervical cancers. Adjuvant chemotherapy and/or radiotherapy are also used in high-risk early stages and in advanced diseases. Hormone therapy, immunotherapy, and targeted therapies (including angiogenesis, kinase and mTOR inhibitors) are also used in metastatic and recurrent diseases [128,129]. To date, no antibody-drug conjugates have been approved for the treatment of both endometrial and cervical cancer. However, several formulations are under clinical trials (Table 3).

### 4.1. ADCs Containing Maytansinoids

FRα is also overexpressed in endometrial tumors. In this case, the overexpression is not as high as in ovarian cancer, but it is still high, with about 64% of endometrial tumors positive for FRα receptors [133]. Because of this, formulations targeting these receptors could be a good therapeutic strategy to get selective chemotherapy in endometrial cancer cells. In fact, mirvetuximab soravtansine, DM-4 conjugated to an anti- FRα-monoclonal antibody (Section 3.1.1), has demonstrated promising anticancer activity in pre-clinical models of this neoplasm [134]. However, its clinical efficacy is not clear. A study, carried out in patients with solid tumors that included patients with endometrial cancer, demonstrated a positive response in 2 of 11 treated patients (18,18%), at doses of 5 mg/kg once every three weeks [83]. This study also included 23 patients with ovarian cancer. In this group, the response was significantly higher and even detected at lower doses (3.3 mg/kg once every three weeks), probably due to the higher levels of FRα in this neoplasm.

Finally, a phase II clinical trial (NCT03835819) is ongoing to evaluate the efficacy of this formulation in combination with prembolizumab in patients with recurrent or persistent endometrial cancer.

With respect to cervical cancer, FRα overexpression has also been related to the carcinogenesis of this neoplasm [135], and FRα targeted formulations could be useful. The aforementioned study also included a woman with this tumor. However, she did not respond to the treatment [83].

### 4.2. ADCs Containing Auristatins

#### 4.2.1. Tisotumab Vedotin

Tisotumab vedotin formulation, MMAE conjugated to an anti-tissue factor monoclonal antibody (Section 3.2.3), has also been evaluated in patients with endometrial and cervical cancer. The dose expansion (phase II) of InnovaTV 201 study included 34 patients with cervical cancer and 14 patients with endometrial cancer. In this study (NCT02001623), the administration of 2 mg/kg once every three weeks showed a positive effect (in terms of tumor size reduction) in 26.5% of the patients with cervical cancer, and response that was significantly higher compared to ovarian cancer patients (response rate of 13.9%). Nevertheless, the response rate in patients with endometrial cancer was very low, just the 7.1% of the patients responded to the treatment [101]. These results indicate that tisotumab vedotin could be good for cervical cancer therapy. In fact, this ADC was also effective in metastatic cervical cancer that did not respond to the previous administration of bevacizumab and doublet chemotherapy, with a similar overall response rate of around 24%. In this study, tissue factor was detected in most of the tumors. However, a correlation between response grade and tissue factor expression was not found [136].

Finally, a phase I-II study in patients with cervical cancer is ongoing to evaluate the efficacy of tisotumab vedotin in combination with bevazizumab, pembrolizumab, and carboplatin (NCT03786081).

#### 4.2.2. A166

A166 is an ADC consisting of an anti-HER-2 monoclonal antibody conjugated to duostatin-5, a MMAF derivative [15,137]. A phase I-II clinical study is underway in patients with HER-2 positive solid tumors, including patients with cervical cancer (NCT03602079). Preliminary results have demonstrated that A166 shows an acceptable safety profile when administered at doses in the range of 0.3–4.8 mg/kg. Ocular toxicities, including dry eye, and vision blurred were common, specially at doses higher than 3.6 mg/kg, appearing in more than 80% of the patients. A positive response, in terms of stable disease and partial response, was detected in almost 60% of the patients. However, these data were calculated in general, and response rates specific to each cancer type have not yet been reported [138].

### 4.3. ADCs Containing Topoisomerase I Inhibitors

Topoisomerase I inhibitors are also good candidates for ADC development, with two formulations: (i) trastuzumab Deruxtecan and (ii) Sacituzumab govitecan, being evaluated in patients with cervical or endometrial carcinomas.

#### 4.3.1. Trastuzumab Deruxtecan

Trastuzumab Deruxtecan, also known as DS-8201a and marketed as Enhertu^®^, is an ADC consisting of an anti-HER-2 monoclonal antibody conjugated to deruxtecan, a topoisomerase I inhibitor through a protease-cleavable linker. It is currently approved by FDA and EMA for the treatment of metastatic breast cancer overexpressing HER-2 receptors [45,46]. It has been demonstrated that the overexpression of HER-2 receptors is associated with invasive endometrial and cervical tumors [41,139], and related to a poor disease outcome, suggesting that they could be a potential target for the development of ADCs.

A phase I study (NCT02564900) undertaken in patients with metastatic advanced tumors, including salivary gland cancer, biliary tract cancer, and endometrial cancer reported that the administration of trastuzumab deruxtecan showed a positive effect. An overall response rate of around 26% and a median progression-free survival of almost one year were detected [140]. However, these promising data were calculated in general for all these tumor types, and no specific rates for endometrial cancer were reported. Currently, several clinical trials are underway to evaluate the efficacy of this formulation as monotherapy in endometrial and cervical cancers among others (NCT04482309) and in combination with olaparib or cesalacertib in endometrial cancer among others (NCT04585958, NCT04704661).

#### 4.3.2. Sacituzumab Govitecan

As aforementioned, SN-38 is a camptothecin analog, specifically is the active metabolite of irinotecan that shows a potent anticancer activity. Due to its severe adverse effects, it cannot be administered as a free drug. Nevertheless, it could be uses as an ADC payload, as the selectivity of these formulations decreases the toxicity of the drug. Sacituzumab govitecan, also known as IMMU-132, and marketed as Trodelvy^®^, has been recently approved, in April 2020, by FDA for the treatment of metastatic triple-negative breast cancer that did not respond to at least two prior therapies for metastatic disease [47,48]. This formulation is being evaluated for the treatment of metastatic breast cancer by EMA under the accelerated assessment category.

This formulation consists of SN-38 conjugated, via a pH cleavable linker, to a monoclonal antibody targeting trophoblastic cell-surface antigen-2 (Trop-2), a transmembrane glycoprotein that participates in the proliferation, survival, and invasion of tumoral cells, and whose overexpression has been related to a poor prognosis [141,142]. Trop-2 is overexpressed in many tumor types including urothelial carcinomas, breast tumors, lung cancers and gynecological cancers. In fact, it has been reported that the overexpression of Trop-2 receptors may participate in the development and pathogenesis of cervical cancer, being a poor prognosis of the disease outcome [143]. Sacituzumab govitecan has demonstrated promising preclinical activity against endometrial and cervical tumors [144,145] and several trials are evaluating its clinical efficacy in these gynecological neoplasms.

In general, this formulation has shown a manageable safety profile when administered at doses of 8 or 10 mg/kg twice every three weeks in patients with epithelial tumors, including ovarian and endometrial cancer, being anemia, nausea, fatigue, diarrhea, alopecia, and neutrophil count decrease the most common adverse effects (NCT01631552) [146]. In the case of endometrial cancer, partial responses have been detected in the 22% of the treated patients [147]. Interestingly, a case report showed a high clinical response (a reduction of the tumor size of around 66% was detected) of this formulation in a patient with recurrent and widespread treatment-resistant uterine serous cancer [148], an aggressive variant of endometrial cancer [149]. These results suggest the potential use of sacituzumab govitecan in endometrial cancer treatment. However, further studies are necessary. A phase 2 clinical trial (NCT04251416) is ongoing to evaluate the efficacy of this ADC as monotherapy in persistent and recurrent endometrial cancer.

### 4.4. ADCs Containing Duocarmycins

Duocarmycins are natural antibiotic metabolites, originally isolated from Streptomyces bacteria that show a potent anticancer activity, with inhibitory concentration 50 values in the picomolar range, becoming promising ADC payloads. They exert their activity by binding the minor groove of the DNA producing an irreversible alkylation of the adenine nucleobases [150,151]. To date, no duocarmycin based ADCs have been approved for cancer therapy, however, several of them are under clinical investigation, including ADCs for the treatment of gynecological cancers.

Trastuzumab duocarmycin, also named SYD985, consists of duocarmycin linked to HER-2 receptors via an enzymatically cleavable linker [130,131]. This formulation has proved an excellent antitumor efficacy in xenograft mouse model of ovarian and also in uterine carcinomas [152,153]. Currently, there are ongoing several clinical trials to evaluate the antitumor response as monotherapy in patients with advanced or metastatic endometrial cancer (NCT04205630) and in combination with niraparib in patients with HER-2 expressing advanced or metastatic endometrial, ovarian or breast tumors (NCT04235101).

### 4.5. BDC-1001

BDC-1001 is an immune-stimulating antibody conjugate, as exerts its anticancer activity stimulating the immune system against the tumor. This formulation consists of a monoclonal antibody targeting HER-2 receptors linked to toll-like receptors (TLR) 7/8 agonists that are antitumor immunomodulatory agents [132]. In fact, Imiquimod (a TLR 7/8 agonist) is approved by FDA for the treatment of non-melanoma skin cancer [154].

A phase I-II clinical trial (NCT04278144) is ongoing to evaluate the efficacy and tolerability and to establish the recommended administration dose of BDC-1001 as monotherapy or in combination with immune checkpoint inhibitors, specifically with pembrolizumab, in patients with HER-2 positive solid tumors, including patients with endometrial cancer [155]. No data have yet been reported for this trial.

## 5. Current Status and Future Perspectives

ADCs represent a great therapeutic option to get a selective action of the antineoplastic agents at the tumoral cells, which considerably reduces the toxicity of these agents and may also increase their antitumor efficacy. In fact, due to the reduction of the side effects, the use of these conjugates makes possible the administration of certain drugs with an excellent antitumor effect but with such high toxicity that cannot be administered as free compounds. These are the cases of the active metabolite of irinotecan (SN-38) and of the auristatins. In the last decade, the development and evaluation of ADCs as anticancer therapy have considerably increased, with a total of 9 ADC formulations approved by FDA and/or EMA for the treatment of different neoplasms, including breast cancer overexpressing HER-2 and triple-negative breast cancer, lymphomas, multiple myeloma, bladder cancer and tumors of the urinary tract. To date, there are no approved ADCs for gynecological malignancies. However, more than 20 ADC formulations have been developed and have reached the clinical stage for these neoplasms, especially for ovarian cancer, the most lethal gynecological tumor, but also for endometrial and cervical cancer.

The ADCs evaluated in ovarian cancer patients target different antigens overexpressed in this neoplasm (Table 2). In general, the ADCs targeting FRα and NaPi2b have reported a promising activity in this type of cancer, especially in advanced stages of the disease and in metastatic tumors, where most parts of these formulations are evaluated, as the current therapies (conventional chemotherapy and other targeted therapies such as PARPs inhibitors) usually fail to treat these disease stages. In all cases, response rates, in terms of partial responses and stable disease, close to or greater than 50% were achieved. Nevertheless, most of the formulations, except Mirvetuximab soravtansine, are at early clinical research (phase I or early phase II studies), and further investigation is needed to confirm their efficacy. On the contrary, the formulations targeted to DPEP3, and ALCAM receptors, showed a low efficacy, with response rates below 10%, indicating that these targets are not the most useful ones for the treatment of this neoplasm. In the case of MUC16, two ADCs, containing MMAE as payload, have been developed: Sofituzumab vedotin and DMUC4064A. Although both use protease cleavable linkers, while DMUC4064A showed good response rates of 45%, Sofituzumab vedotin showed a low efficacy of 7%, suggesting the importance of the conjugation site and method

In the case of endometrial and cervical cancers, FRα, HER-2, tissue factor and Trop-2 receptors (the latter only in endometrial cancer) are the exploited targets (Table 2). Of all of them, HER-2 targeted formulations, especially A166 ADC, are the most promising formulations for endometrial cancer, with responses rates greater than 50%. In this neoplasm, targeting FRα does not seems to be as effective as in ovarian cancer, with responce rates of around 30% probably due to the lower expression of FRα in endometrial cancer. In the case of cervical tumors, lower response rates (around or below 25%) were obtained, with the best-reported results corresponding to Tisotumab vedotin, an ADC targeting tissue factor. Interestingly, this formulation exhibited lower response rates in both endometrial (≈13%) and ovarian cancer patients (≈7%), suggesting that it could have a higher utility in the treatment of cervical cancer. However, it should be considered that the antibodies evaluated in patients with endometrial and cervical carninomas are at early research stages and further investigation is needed, especially considering that many of the explained studies were carried out in patients with different cancer types, with a limited number of women with these carcinomas, and the reported response rates are not specific for each cancer type.

Among all listed formulations for gynecological cancer treatment, Mirvetuximab soravtansine that consists of DM-4 conjugated to an anti-FRα monoclonal antibody is the most advanced formulation, with phase III clinical studies. This formulation, at doses of 5–6 mg/kg, showed excellent results in ovarian cancer not only as monotherapy but also in combination with bevacizumab and carboplatin. This is particularly interesting, considering that this is difficult to treat neoplasm due to its aggressiveness and late diagnosis.

Although there are currently no ADCs approved for the treatment of gynecological cancers, it is expected that in the near future the number of formulations evaluated and approved for the treatment of these neoplasms, especially for the treatment of ovarian cancer, will increase significantly, with formulations incorporating antibodies with an antitumor effect per se being of particular interest.

## Figures and Tables

**Table 1:** Targets and payloads exploited in the ADCs evaluated in patients with ovarian, endometrial, and cervical cancer.

Cancer Type	ADC	Targets	Payloads	Reference
Ovarian cancer	Mirvetuximab soravtansine	Folate Receptor α	DM-4 (Maytansinoid)	[26,27]
	Praluzatamab ravtansine	ALCAM/CD116	DM-4 (Maytansinoid)	[28]
	Anetumab ravtansine	Mesothelin	DM-4 (Maytansinoid)	[29,30]
	DMUC4064A	MUC-16	MMAE (Auristatin)	[31]
	Sofituzumab vedotin	MUC-16	MMAE (Auristatin)	[32]
	Tisotumab vedotin	Tissue Factor	MMAE (Auristatin)	[33]
	Enapotamab vedotin	AXL	MMAE (Auristatin)	[34]
	Lifastuzumab Vedotin	NaPi2b	MMAE (Auristatin)	[35]
	Cofetuzumab pelidotin	Protein Tyrosine Kinase 7	Auristatin-0101	[36]
	Upifitamab rilsodotin	NaPi2b	MMAF (Auristatin)	[37,38]
	PF-06647263	EFNA-4	Calicheamicin	[39]
	Tamrintamab pamozirin	Dipeptidase 3	Pyrrolobenzodiazepine dimer	[40]
	SC-004	Claudin 6 and 9	Pyrrolobenzodiazepine dimer	[41]
	STRO-002	Folate Receptor α	SC209 (Hemisaterlin derivate)	[42]
	MORAB-202	Folate Receptor α	Eribulin	[43]
Endometrial cancer	Mirvetuximab soravtansine	Folate Receptor α	DM-4 (Maytansinoid)	[26,44]
	Tisotumab vedotin	Tissue Factor	MMAE (Auristatin)	[33]
	A166	HER-2	Duolastin (MMAFderivate)	[15]
	Trastuzumab Deruxtecan	HER-2	Deruxtecan	[45,46]
	Sacituzumab govitecan	Trop-2	SN-38	[47,48]
	Trastuzumab duocarmycin	HER-2	Duocarmycin	[33]
Cervical cancer	Mirvetuximab soravtansine	Folate Receptor α	DM-4 (Maytansinoid)	[26,27]
	Tisotumab vedotin	Tissue Factor	MMAE (Auristatin)	[33]

ALCAM: activated leukocyte cell adhesion molecule, EFNA-4: tyrosine kinase ephrin receptor A4, HER-2: Human epidermal growth factor receptor 2, MMAE/MMAF: Monomethyl auristatin E/F, NaPi2b: sodium-dependent phosphate transport protein 2B, Trop-2 Trophoblast cell surface antigen 2.

**Table 2:** Structure of the ADCs developed for ovarian cancer therapy.

Ovarian Cancer					
ADCs containing maytansinoids	**Formulations**	**Antineoplastic Drug**	**Target**	**Linker**	**References**
	Mirvetuximab soravtansine	DM4	FRα	Cleavable disulphide linker	[26,27]
	Praluzatamab ravtansine		ALCAM/CD116		[28]
	Anetumab ravtansine		MESOTHELIN		[29,30]
ADCs containing auristatins	DMUC4064A	MMAE	MUC-16	Protease cleavable linker	[31]
	Sofituzumab vedotin				[32]
	Tisotumab vedotin		Tissue Factor		[33]
	DMOT4039A				[79]
	Enapotamab vedotin		AXL		[34]
	Lifastuzumab Vedotin		NaPi2b		[35]
	Cofetuzumab pelidotin	Auristatin-0101	PTK-7		[36]
	Upifitamab rilsodotin	MMAF	NaPi2b	------------	[37,38]
ADCs containing calicheamicins	PF-06647263	Calicheamicin	EFNA-4	Hydrazone cleavable linker	[39]
ADCs containing pyrrolobenzodiazepines	Tamrintamab pamozirin	Pyrrolobenzodiazepine	DPEP3	Protease cleavable linker	[40]
	SC-004		CLDN6/9	------------	[41]
ADCs containing hemiasterlin derivates	STRO-002	SC209	FRα	Protease cleavable linker	[42]
ADCs containing eribulin	MORAB-202	Eribulin	FRα	Protease cleavable linker	[43]

ALCAM: activated leukocyte cell adhesion molecule, CLDN6/9: Claudin 6/9, DPEP3: Dipeptidase 3, EFNA-4: tyrosine kinase ephrin receptor A4, FRα: Folate receptor α, MMAE/MMAF: Monomethyl auristatin E/F, MUC-16: Mucin 16, NaPi2b: sodium-dependent phosphate transport protein 2B, PTK-7: Tyrosine-protein kinase-like 7.

**Table 3:** Structure of the ADCs developed for endometrial and cervical cancer therapy.

Endometrial and Cervical Cancer					
ADCs containing maytansinoids	**Formulations**	**Antineoplastic Drug**	**Target**	**Linker**	**Reference**
	Mirvetuximab soravtansine	DM4	FRα	Cleavable disulphide linker	[26,44]
ADCs containing auristatins	Tisotumab vedotin	MMAE	Tissue Factor	Protease cleavable linker	[33]
	A166	Duostatin-5 (MMAF derivate)	HER-2	--------------	[15]
ADCs containing topoisomerase I inhibitors	Trastuzumab Deruxtecan	Deruxtecan	HER-2	Protease cleavable linker	[45,46]
	Sacituzumab govitecan	SN-38	Trop-2	pH cleavable linker	[47,48]
ADCs containing duocarmycins	Trastuzumab duocarmycin	Duocarmycin	HER-2	Protease cleavable linker	[130,131]
ADCs containing inmmunomodulatory agents	BDC-1001	TLR 7/8	HER-2	---------------	[38,132]
